# Zinc oxide-decorated polypyrrole/chitosan bionanocomposites with enhanced photocatalytic, antibacterial and anticancer performance†

**DOI:** 10.1039/c9ra06493a

**Published:** 2019-12-12

**Authors:** Nafees Ahmad, Saima Sultana, Syed Mohd Faisal, Anees Ahmed, Suhail Sabir, Mohammad Zain Khan

**Affiliations:** Environmental Research Laboratory, Department of Chemistry, Aligarh Muslim University Aligarh 202002 India dr_mzain.fa@amu.ac.in; Molecular Immunology Laboratory, Interdisciplinary Biotechnology Unit, Aligarh Muslim University Aligarh 202002 India; Hybridoma Laboratory, National Institute of Immunology Aruna Asaf Ali Marg New Delhi 110067 India

## Abstract

A bio-nanocomposite matrix of polypyrrole grafted ZnO/chitosan (Ppy/C/Z) was synthesized *via* the *in situ* polymerization of pyrrole with different weight fractions of ZnO. Incorporation of ZnO nanoparticles with polypyrrole enhances the photocatalytic, antibacterial as well as cytotoxic properties of the resultant composite. Characterizations of the synthesized product were performed by X-ray diffraction (XRD), Fourier transform infrared spectroscopy (FTIR) and thermal analysis (TGA and DTA). Surface morphology and particle size were determined by SEM and TEM. The elemental composition of the material was studied by EDX coupled with SEM. Electrochemical surface area was calculated from electrochemical double layer capacitance (EDLC) measurements using cyclic voltammetry. The photocatalytic activity of the composite material was tested by monitoring the degradation of reactive orange-16 (RO-16), Coomassie Brilliant Blue R-250 (CBB-R-250) and Methylene Blue (MB) dyes and the composite was found to be an effective catalyst in the presence of a UV light source. Various scavengers were used to detect the reactive species involved in the photocatalytic process. Furthermore, the stability of the photocatalyst was assessed by recycling experiments. Moreover, the Ppy/C/Z bio-nanocomposite shows potential application with anti-bacterial and anti-cancer activity against Gram-positive and Gram-negative bacterial pathogens and human cancer cell lines (HeLa and MCF-7). The experimental data confirm that the bio-nanocomposite of Ppy/C/Z showed excellent anti-bacterial and anti-cancer activity as compared to a pristine polypyrrole and chitosan formulation (Ppy/C). The apoptosis data with varying concentrations of Ppy/C/Z reveal the remarkable activity against these cancer cell lines.

## Abstract 2

Bio-nanocomposites were synthesized *via* grafting polypyrrole/ZnO onto chitosan chain for the photodegradation of organic pollutants and biomedical applications.

## Graphical abstract

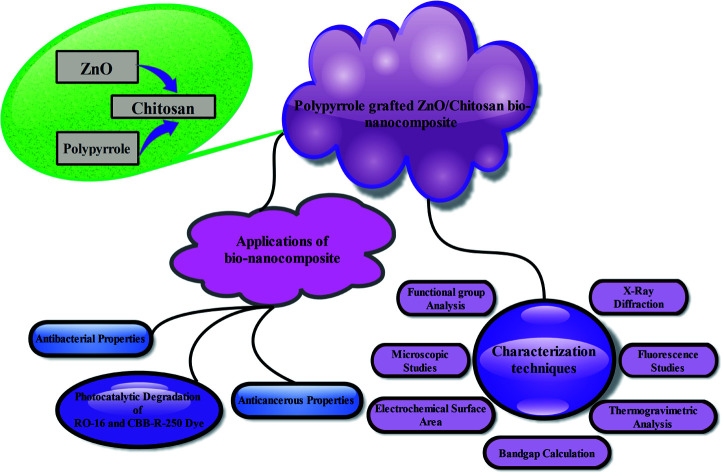

## 1. Introduction

Nanoparticles have been attributed with their remarkable and unique properties almost in every field, *e.g.*, automobiles, sensors, environment and biomedicine. Nanoparticles due to their large surface area become more effective in the catalytic process. Its activity and efficiency can be enhanced by doping with other nanoparticles or conducting polymers in the form of nanocomposites or polymer nanocomposites, respectively.^1^ Nanotechnology has been the most promising tool in the biomedical field in applications including dental implants, tissue engineering, drug delivery, cancer treatment and antibacterial treatment.^2^ For the past few decades, nanocomposites have also been used as effective photocatalysts for the degradation of organic pollutants, such as, dyes, drugs, and pesticides, as these organic pollutants are toxic, carcinogenic and hazardous in nature and have been the major sources of water pollution.

Among the organic pollutants, dyestuffs from the textile and food industries are directly discharged into the water bodies, thus causing severe water and environmental pollution.^3^ Due to its stability against light and temperature, treatment of dyes has now become a prominent issue since dyes cannot be removed from the water bodies by the simple treatment of water.^4^ For the past few decades, nanocomposites have attracted great attention due to their remarkable and unique properties, due to which they can be applied for the photodegradation of the dyes.^5^ Semiconductor nanoparticles such as BiVO_4,_ ZnS, CdS, WO_3_, TiO_2_, Sb_2_S_3_, and Bi_2_S_3_ have been extensively used as photocatalysts for the degradation of dyed.^6–8^ ZnO nanoparticles with semiconducting properties, absorption properties, high surface area, good thermal stability and ease of processing are suitable for the purpose of photodegradation.^9,10^ These nanoparticles upon the absorption of the light generate charges carriers (e^−^ and h^+^) that are helpful in the degradation of toxic organics.

Furthermore, conducting polymers such as polyaniline (PANI), polypyrrole (Ppy), and polythiophene (PTh) have been effectively applied in the last few years for the photocatalytic degradation of organic pollutants due to their high surface area, redox ability and tunable bandgap. Moreover, these conducting polymers have found many applications such as in gas sensors, biosensors, electronic displays and many electronic devices^11^ due to their π-electron conjugate system and electron hole mobility. Among these conducting polymers, polypyrrole (Ppy) has been extensively investigated due to its heterocyclic structure,^12^ non-toxicity,^1^ ease of synthesis, and high conductivity. Ppy can also be used as a catalytic support in electrochemical fuel cells, drug delivery, sensors, and batteries.^13,14^ Electrochemically prepared polypyrrole/chitosan composites have already been reported for applications as electrochemical biosensors and anti-corrosive coatings,^15^ and also applied as surface protective agents to improve the performance of 316L SS bio-implants, as reported by Kumar *et al.*^16^ With regards to photocatalytic activity, polypyrrole exhibited a synergistic effect with ZnO to enhance the activity of the photocatalyst by minimizing the recombination behaviour of the charge carriers.

Another approach investigated in order to improve the efficiency and activity of nanocomposites is introducing biopolymers, such as chitosan, to form bio-nanocomposites. chitosan is a linear macromolecular polysaccharide and the second most abundant biopolymer after cellulose.^17–19^ Being a biodegradable polymer with some unique properties, it finds many applications in different field such as medicine, food,^20^ drugs,^21^ and environmental remediation.^22^ Chitosan has –NH_2_ groups that convert into NH_3_^+^ during protonation, which attack the anionic groups and remove those parts from the dye. In addition to its property to act as a photocatalyst for the degradation of the organic pollutants, it can also be applied for the treatment of the cancer as well as bacterial infections. Predominantly, it is a well-recognized fact that bio-nanocomposites of chitosan induce cytotoxicity in the cancer cells, but not in healthy cells. In addition to this, bio-nanocomposites of chitosan have effective and efficacious anti-microbial potential.^23^

To check our hypotheses, the anti-bacterial and anti-cancer potential of Ppy/C/Z was evaluated against Gram-positive and Gram-negative bacterial pathogens and human cancer cell lines (HeLa and MCF-7). Efforts were made to prepare polypyrrole-grafted chitosan bionanocomposites with different weight percentage of the ZnO nanoparticles. The concentration of nanoparticles, oxidant to monomer ratio and interfacial interactions of the matrices greatly affect the activity of nanocomposites.^24^ Research based on the nanocomposite matrices of polypyrrole, chitosan and ZnO as efficient photocatalysts for the degradation of the RO-16 dye is not reported in literature. However, the hybrid structure of polypyrrole and chitosan has been applied for biomedical and electroanalytical applications.^25,26^ In the present study, bionanocomposites were synthesized by grafting polypyrrole using pyrrole monomer and FeCl_3_ as the oxidant on chitosan as the backbone with the incorporation of ZnO nanoparticles.^27^ Different weight fractions of the ZnO nanoparticles were introduced to Ppy/chitosan to study the effect of the nanoparticles under UV irradiation for the photodegradation of the RO-16 dye.

## 2. Experimental

### 2.1. Materials and reagents

All reagents were of analytical grade (AR) and supplied by Fischer Scientific. Zinc chloride, sodium hydroxide, chitosan, ferric chloride, sodium lauryl sulphate (SLS), pyrrole and methanol were used as received. Agarose, low melting point agarose (LMPA), Triton X-100, Trypan blue and MTT were purchased from Sigma Aldrich (St. Louis, MO). Tissue culture-grade plastic-ware was purchased from Eppendorf, Hamburg, Germany. Tissues and cells were maintained in Roswell Park Memorial Institute (RPMI)-1640 culture medium supplemented with 10% fetal bovine serum (FBS) (Gibco, life technologies), and 0.5% antibiotic antimycotic solution (Sigma-Aldrich Co, St Louis, MO, USA) at 37 °C in a humidified incubator maintaining 5% CO_2_. Bacterial culture media Luria Bertani, agar and brain heart infusion were procured from Himedia Laboratories Pvt. Ltd. All other chemicals were of analytical grade and used without further purification.

### 2.2. Synthesis of biopolymer nanocomposites

#### 2.2.1. Synthesis of ZnO nanoparticle

Zinc oxide nanoparticles were prepared by the direct precipitation method using ZnCl_2_ and NaOH as precursors. Briefly, 0.2 M ZnCl_2_ was prepared in distilled water and equal volume of 0.2 M NaOH was added dropwise to this solution under constant stirring. The samples were kept under constant stirring on a magnetic stirrer for about 2 h. Then, a white precipitate of ZnO nanoparticles was obtained, which was then filtered and dried at 100 °C for 2 h.^27^

#### 2.2.2. Synthesis of ZnO-chitosan nanocomposite

Different weight fractions of ZnO-chitosan were prepared by adding different weight percent of ZnO (*i.e.*, 10%, 20%, 30%, and 40%) to 1 mM chitosan solution (100 ml) prepared in 1% (v/v) acetic acid solution. The solution was homogenized in an ultrasonicator for about 30 min.

#### 2.2.3. Synthesis of polypyrrole grafted ZnO-chitosan nanocomposites

The synthesis of Ppy-grafted ZnO-chitosan bionanocomposites was performed in an inert atmosphere by passing nitrogen gas in a beaker covered from top and containing 1 mg ZnO-chitosan in 75 ml acetic acid (1% solution). To this solution, 0.18 mol of pyrrole monomer dissolved in 50 ml of acetic acid at a temperature of 0–10 °C. After 1 h of mixing, FeCl_3_ solution (prepared in acetic acid) was added dropwise to the above mixture so as to initiate the chemical oxidative polymerization of pyrrole followed by stirring for about 16 h. A black coloured product was obtained after 16 h, filtered and dried at 100 °C for 2 h.^28^

### 2.3. Characterization

Fourier transform infrared (FTIR, PerkinElmer spectrum 2) spectroscopy was performed and the samples were scanned at room temperature in the region 450–4000 cm^−1^ for the identification of various characteristic functional groups. The polymer bio-nanocomposites were dispersed in KBr and compressed into pellets before characterization. The crystal structure and particle size of the as-prepared bio-nanocomposites were studied and confirmed by using the powder X-ray diffraction technique (Bruker D8 Advance) with Cu Kα radiation. The powder scanning was performed in at 2*θ* values ranging from 10° to 80°. The surface morphology, size and chemical composition of the as-prepared bio-nanocomposites were studied by using SEM (scanning electron microscopy) (Joel JSM-6360) and TEM (transmission electron microscopy) coupled with EDX (energy dispersive X-ray spectroscopy). UV-Visible spectroscopy (Thermo scientific Evolution 201) was performed to check the absorbance of the degraded aliquots from the reactor and the recombination rate of the photo-induced charge carriers was examined by measuring photoluminescence intensity *via* fluorescence spectroscopy (Hitachi F-2500) at an excitation wavelength of 320 nm. The electrochemical surface area of the materials was investigated using CV (cyclic voltammetry) (Auto lab 204 Netherland).

### 2.4. Sample fabrication for cyclic voltammetry

**Fig. 1:**
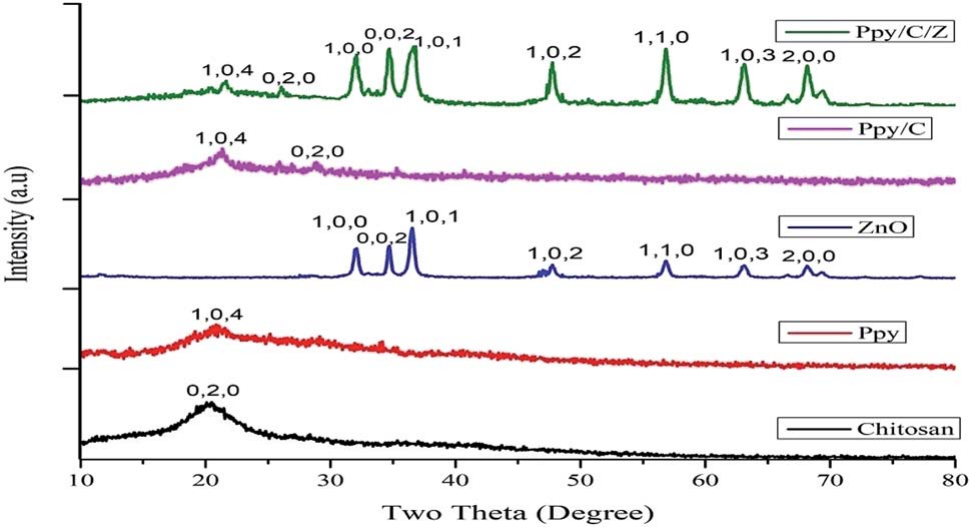
X-ray diffractograms of the as-prepared nanocomposite materials.

The electrochemical nature of the as-prepared bio-nanocomposites was studied by CV. From the cyclic voltammogram data, electrochemical surface area (ECSA) was calculated by determining the electrical double layer capacitance (EDLC). CV was performed using a three-electrode system in the applied potential range of −1.5 to +1.5 V. Ag/AgCl was used as the reference electrode, where the concentration of Cl^−^ in the Ag/AgCl electrode was 3 M and the reference potential of Ag/AgCl was 0.193 mV. A platinum (Pt) wire was taken as the counter electrode and glassy carbon electrode (GCE) was used as the working electrode, while 0.1 M solution of KOH was used as an electrolyte. Prior to analysis, the sample was purged with nitrogen in order to maintain the inert atmosphere. The sample fabrication on the GCE surface was done in accordance to Saquib *et al.*^29^ Typically, 0.2 mg of each photocatalyst sample was taken along with 5 μl isopropyl alcohol and H_2_O, and chitosan solution (with glacial acetic acid) was used as the binder. The sample was fabricated on the GCE surface by the dip-casting method and kept for drying in open air for 30 min. A pictorial diagram of the three-electrode system (CV) used in the present study is shown in Fig. 1S (ESI†).

### 2.5. Evaluation of the photocatalytic activity and study of reactive species (scavengers)

The photocatalytic evaluation of the as-prepared bio-nanocomposite material has been investigated by monitoring the degradation of RO-16, CBB-R-250 and MB dyes. The experiment was performed in a photochemical reactor made up of glass equipped with a UV lamp, and an oxygen pump was used to supply atmospheric oxygen to the sample. The temperature was maintained at 25 °C. Further, 0.3 g of photocatalyst was taken against 300 ml of the dye sample. Before the irradiation, the dye solution was stirred in absence of UV light to attain adsorption–desorption equilibrium between the dye and photocatalyst. After the equilibrium was attained, 5 ml aliquots were taken out of the reactor on regular intervals and the degradation of the RO-16 dye was measured as a function of irradiation time against absorbance at maximum absorption wavelength of 493 nm for RO-16. The degradation efficiency was calculated by using the following eqn (1):1
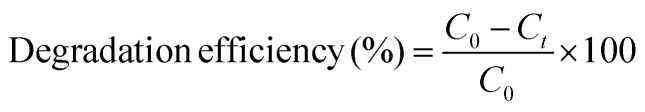
where *C*_0_ is the initial concentration and *C*_*t*_ is the concentration at time ‘*t*’.

The stability of the photocatalyst is very important for its practical use, so to check the stability of the bio-nanocomposites, the photodegradation experiment was repeated four times. The photocatalyst after the first cycle was recovered through centrifugation, washed with acetone and distilled water, dried and used for the next cycle. Further, to assure the generation of reactive species during photocatalytic degradation, various scavengers, namely, *p*-benzoquinone, disodium ethylenediaminetetraacetate (EDTA) and isopropyl alcohol (IPA) were used for the detection of ·O_2_^−^, h^+^ and ·OH, respectively. Accordingly, 2 mM of each scavenger was added to the aqueous solution of the RO-16 dye before adding the photocatalyst so as to identify reactive species.

### 2.6. Bacterial strains

Bacterial strains were used for screening Gram-negative *Escherichia coli* (American Type Culture Collection (ATCC)® 25922™; ATCC, Manassas, VA, USA) and Gram-positive *Staphylococcus aureus* (ATCC 25923). *E. coli* strains were cultured in Luria Bertani broth, while *S. aureus* were cultured in brain heart Infusion broth medium for 12–18 h at 37 °C.

### 2.7. Cell culture

Cell lines used in this study were procured from the Cell Repository of the National Centre for Cell Science, Pune (India). Human cervical cancer cell line (HeLa) and human breast cancer cell line (MCF-7) were used in the present study and cultured in RPMI 1640 supplemented with 10% heat-inactivated FBS and 0.5% antibiotic-antimycotic solution. Cells were maintained at 37 °C in a humidified incubator providing 5% CO_2_.

### 2.8. Minimum inhibitory concentration (MIC) and minimum bactericidal concentration (MBC) evaluation

Broth dilution assay was performed by inoculating 1 × 10^6^ cells of various bacterial strains in each well. The serial dilutions of the as-synthesized bio-nanocomposites prepared in sterile broth culture media were then added and incubated for 24 h at 37 °C in the shaking incubator following a published protocol by Andrews *et al.*^30^ Bacterial growth was assessed based on the broth's turbidity, where the absence of turbidity was an indicator of successful antimicrobial sensitivity. The lowest concentration where the broth is clear indicates the minimum inhibitory concentration of various formulations that can successfully inhibit bacterial growth. The lowest concentration of substance that will prevent the growth of bacteria after sub-culturing on to the antibiotic free media is called the MBC of that antimicrobial substance.

### 2.9. Agar well diffusion assay

The antibacterial potential of the as-synthesized chitosan and polypyrrole-based bio-nanocomposites was assessed against *E. coli* and *S. aureus* using the agar well diffusion method following a published protocol, as standardized in our laboratory.^31,32^ The inoculum was prepared by diluting the overnight cultures with sterile normal saline to a 0.5 McFarland standard. The agar Petri plates were prepared by spreading 1 × 10^7^ CFU per 50 μl of the mature broth culture of specific bacterial strains with a sterile L-shaped glass rod. An 8 mm well was created in each Petri-plate with the help of sterile yellow tips. Bio-nanocomposites were then suspended in sterile phosphate buffer saline and used to evaluate its antibacterial properties. Experimental procedures were performed under sterile conditions using bio-safety level 2 (BSL-2) hoods and Petri plates that were incubated at 37 °C for 24 h. The sensitivity and efficacy of the as-synthesized bio-nanocomposites were determined on the basis of the diameter of the zone of inhibition against human pathogenic strains. The inhibition zone was measured in triplicate with various chitosan-based bio-nanocomposites; average of the three values was calculated for the antibacterial activity.

### 2.10. Evaluation of the cell cytotoxicity of the as synthesized Ppy/C and Ppy/C/Z by performing the MTT assay

The anticancer potential of the chitosan and polypyrrole-based bio-nanocomposite was determined using the 3-(4,5-dimethylthiazol-2-yl)-2,5-diphenyltetrazolium bromide (MTT) assay following a previous report.^33^ MTT assay is a colorimetric assay, which is based on the ability to cleave the tetrazolium ring of the MTT by the mitochondrial dehydrogenase enzyme of viable cells to form insoluble formazan crystals, which deposit on the living cells. This insoluble formazan is then dissolved by the addition of a suitable solvent to form a purple coloured solution. The amount of formazan crystals obtained is directly proportional to the number of living cells. The absorbance of this coloured solution was measured at 570 nm using GMB-580 Automatic Microplate Reader (Genetix Biotech Asia Pvt. Ltd.).

### 2.11. Apoptosis assay by employing flow cytometry

The human cervical cancer cell line (HeLa) was used in the current study. HeLa cells were cultured in Roswell Park Memorial Institute (RPMI)-1640 supplemented with 10% heat-inactivated fetal bovine serum with 0.5% penicillin/streptomycin maintained in a humidified incubator at 37 °C with 5% CO_2_. 1 × 10^6^ cells per well were seeded in the 96-well plate. After 24 h of incubation, media were changed with 100 μl of fresh media containing the indicated concentration of the Ppy/C/Z bio-nanocomposite. After 24 h treatment with the bio-nanocomposites, cells were harvested and washed twice with DPBS. Cells were then resuspended in annexin V binding buffer containing propidium iodide and FITC-annexin V and incubated for 15 min at RT in the dark. FACS buffer was then added, and cell suspensions were analysed by flow cytometry. Treated cells were analysed on a BD verse flow cytometry instrument employing flow Jo software. Live cells are double negative, late apoptotic cells are double positive, while early apoptotic cells are annexin V-positive and PI-negative. The total apoptotic cells were represented as Annexin V-positive cells.

## 3. Results and discussion

### 3.1. X-ray diffractograms

The powder XRD pattern of pure compounds and bionanocomposite matrices are shown in Fig. 1. Characteristic diffraction peaks of Ppy/C/Z were detected at 2*θ* angles of 21.5°, 26.07°, 32.2°, 34.7°, 36.7°, 37.9°, 55.9°, 66.09°, and 71.1°, corresponding to *hkl* values of (104), (020), (100), (002), (101), (102), (110), (103), and (200). The characteristic diffraction peaks of Ppy/C were detected at 2*θ* values of 21.4° and 28.6°, corresponding to *hkl* values of (104) and (020). The pattern of chitosan shows *hkl* values of (020) corresponding to a 2*θ* value of 21.3°,^34^ while that of Ppy shows *hkl* values of (104) at a 2*θ* value of around 22.7°, which are in accordance with the findings reported by Li *et al.*^35^ The diffraction peaks of ZnO were detected at 2*θ* values of 32.2°, 34.6°, 36.5°, 47.7°, 56.7°, 63.1°, and 68.2° corresponding to *hkl* values of (100), (002), (101), (102), (110), (103), and (200).^36^ The spectral peaks of chitosan and Ppy in the pattern of Ppy/C/Z have been shifted slightly from their actual positions, probably due to the dislocation effect upon incorporation.^37^ The sharp diffraction peaks at the abovementioned angles indicate the crystalline behaviour of the bio-nanocomposite. In the diffraction patterns of bio-nanocomposites, all the constituents, *i.e.*, polypyrrole, chitosan and ZnO were detected and from the pattern, the formation of bionanocomposites was confirmed.^38,39^ The average crystal size of the nanocomposites was calculated using Scherrer's formula (eqn (2)):2
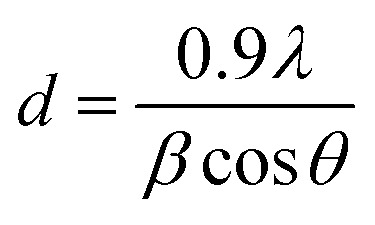
where *d* is the average crystal size, *λ* is the X-ray wavelength, *β* is the full width of the half maximum calculated in radians, and *θ* is the Bragg's angle of the diffraction peaks. The average crystal size calculated for the prepared Ppy/C/Z nanocomposites was found to be 17.215 nm.

### 3.2. FTIR analysis

**Fig. 2:**
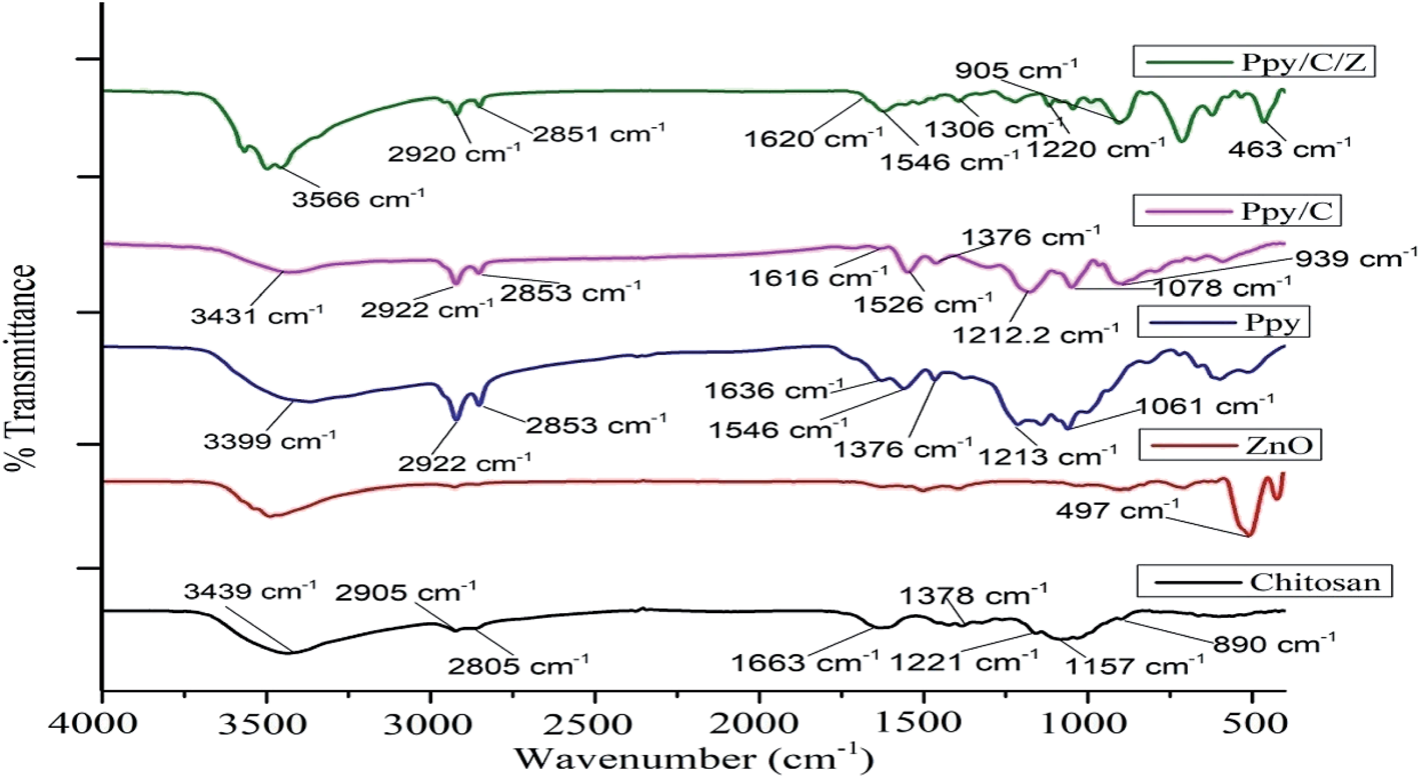
FTIR spectra of the as-prepared nanocomposite materials.

The functional group analysis of the as-prepared bio-nanocomposites was performed using FT-IR. Fig. 2 represents the FT-IR spectra of Ppy/C as well as Ppy/C/Z. Characteristic adsorption peaks of Zn–O are observed at around 497 cm^−1^, as reported elsewhere.^40^ The broad band at 1636 and 1548 cm^−1^ show the stretching vibration of C=C and C–C in the pyrrole ring, respectively.^41^ The spectrum of polypyrrole shows characteristic C–H in-plane and C–N stretching vibrations at 1376 cm^−1^ and 1213 cm^−1^, respectively.^42^ The peak observed at 3399 cm^−1^ is due the N–H functional group. The characteristic O–H stretching peak of chitosan is obtained at around 3439 cm^−1^, and the peak at 2905 cm^−1^ is ascribed to axial stretching of the C–H bond, while the peak at 1663 cm^−1^ is ascribed to the amide group.^43^ Peaks at 1221 cm^−1^ and 1378 cm^−1^ are attributed to C–N stretching and N–H angular deformation, and peaks appearing at 1157  cm^−1^ and 890 cm^−1^ correspond to C–O and C–O–C stretching vibrations of chitosan, respectively.^44^ However, in case of the spectra of Ppy/C/Z bio-nanocomposites, there is a slight change in the peak positions of the vibrations. Pure ZnO shows the Zn–O vibration at 497 cm^−1^, but upon the incorporation to Ppy, the peak shifted towards the lower region at 463 cm^−1^ and the O–H peaks of chitosan shifted towards the higher region from 3439 cm^−1^ to 3497 cm^−1^.

### 3.3. Microscopy studies *via* SEM, EDX and TEM

**Fig. 3:**
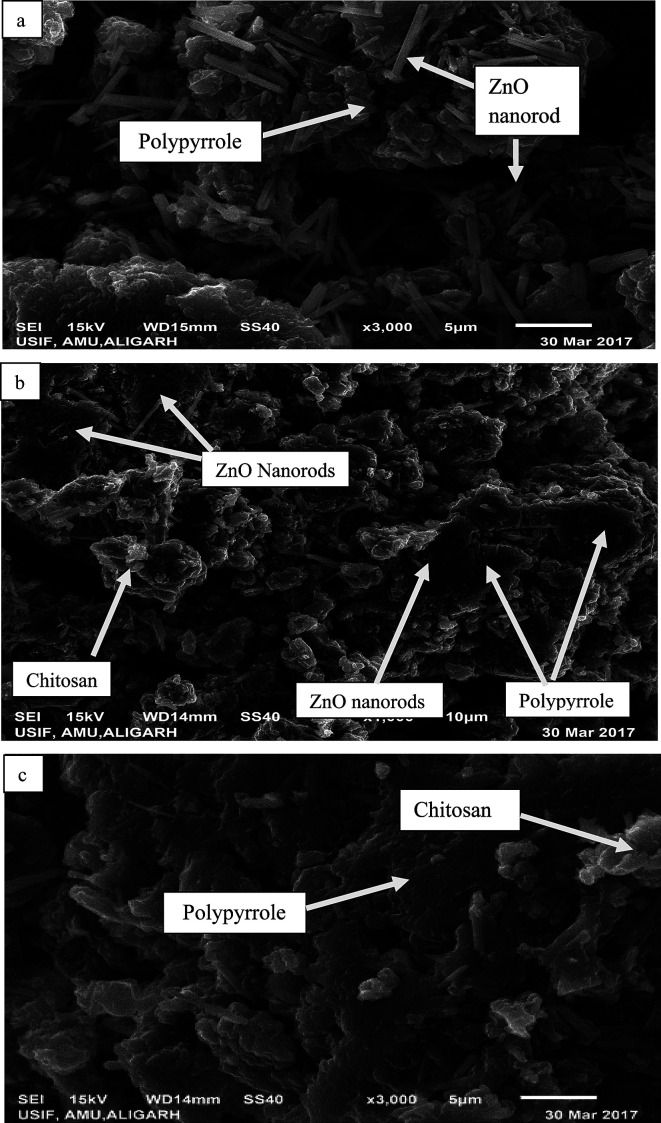
SEM images of the as-prepared nanoparticles and nanocomposites: (a) Ppy/ZnO (b) Ppy/chitosan/ZnO and (c) Ppy/chitosan.

The surface morphology of the as-prepared bio-nanocomposites was studied *via* SEM, whereas particle size and elemental composition of the as-synthesized bio-nanocomposites were studied *via* TEM and EDX analysis, respectively. As shown in Fig. 3a, the presence of ZnO nanoparticles doped on Ppy can be observed, in which ZnO nanorods are scattered over the surface of the Ppy.^45^ The matrices of ZnO, chitosan and polypyrrole are mixed in an irregular fashion, as shown in Fig. 3b. The microscopic particles of ZnO are clearly seen to be adsorbed and dispersed on the surface of Ppy in an irregular fashion.^46^ The polymer matrices of the nanocomposites, polypyrrole and chitosan are mixed, as shown in Fig. 3c.

**Fig. 4:**
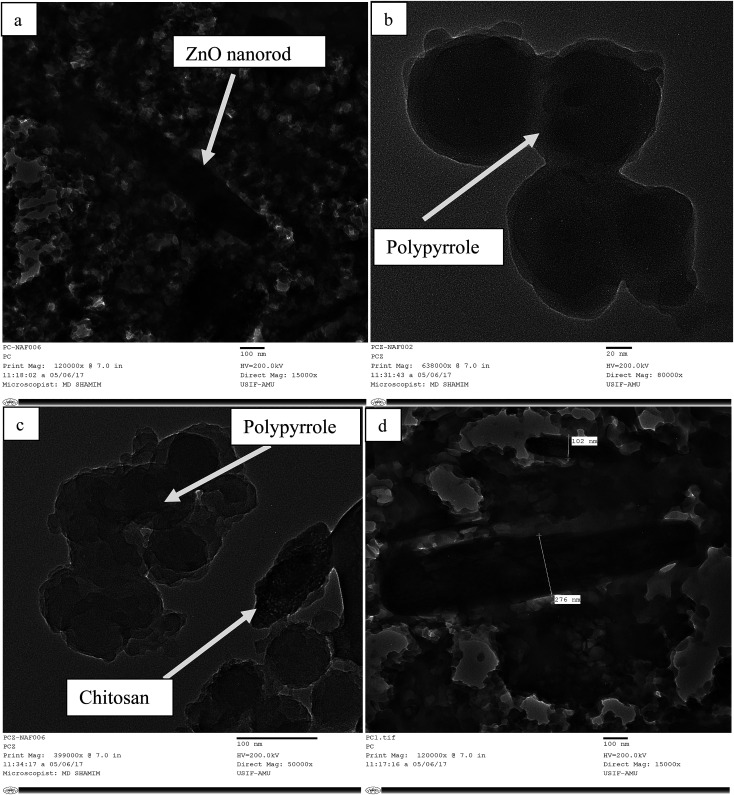
TEM micrographs of the as-prepared nanoparticles and nanocomposites: (a) Ppy/ZnO (b) Ppy (c) Ppy/chitosan and (d) Ppy/C/ZnO.

TEM micrographs of the matrix show the particle size of the nanocomposites, which is in the range of 10–100 nm. In Fig. 4a, the nanorods of ZnO are shown to be uniformly mixed with Ppy. Fig. 4b and c are the TEM images of pure Ppy, and two different phases of Ppy and chitosan can easily be seen. ZnO nanorods are easily dispersed over the polypyrrole surface due to its porous structure. The grain size of Ppy increased upon the incorporation of ZnO as compared with pure Ppy and chitosan, as shown in Fig. 4d.^9^ The elemental composition and purity of the bionanocomposites can be determined with the help of EDX analysis in Fig. 2S (ESI†).

### 3.4. Thermal analysis

Thermal analysis of the as-prepared bio-nanocomposites was performed using TGA and DTA under nitrogen atmosphere. The TGA and DTA curves of Ppy/C and Ppy/C/Z are shown in Fig. 3S (ESI†). As shown in the figure, the incorporation ZnO to the Ppy/C matrix slightly affects the thermal stability of the bio-nanocomposite matrices. The strong endothermic peak in the range 200–300 °C corresponds to a TGA weight loss of the bio-nanocomposites in the region from 50 to 300 °C, which is due to the evaporation of the physically bound water adsorbed on the surface due to the hygroscopic nature of the polypyrrole.^47^ The exothermic peaks in range 300–500 °C in the DTA curve results due to the weight loss in the range 250–500 °C in TGA curve, which is due to the removal of lattice water. Finally, the rapid weight loss above 500 °C is due to the degradation of the polymeric part of the matrix, corresponding to the exothermic peak of DTA in the range 500–700 °C. The weight loss observed in Ppy/C at mid-point (163.57 °C, 247.83 °C and 468.72 °C) was found to be 2.116, 3.371 and 1.822 mg, respectively, where as in the case of Ppy/C/Z, weight loss observed at the midpoint (189.66 °C, 259.59 °C and 522.48 °C), and was found to be 2.497, 2.154 and 1.41 mg, respectively, in temperature ranges of 50–250 °C, 250–450 °C and 450–800 °C, respectively.^48^

### 3.5. UV-DRS analysis

**Fig. 5:**
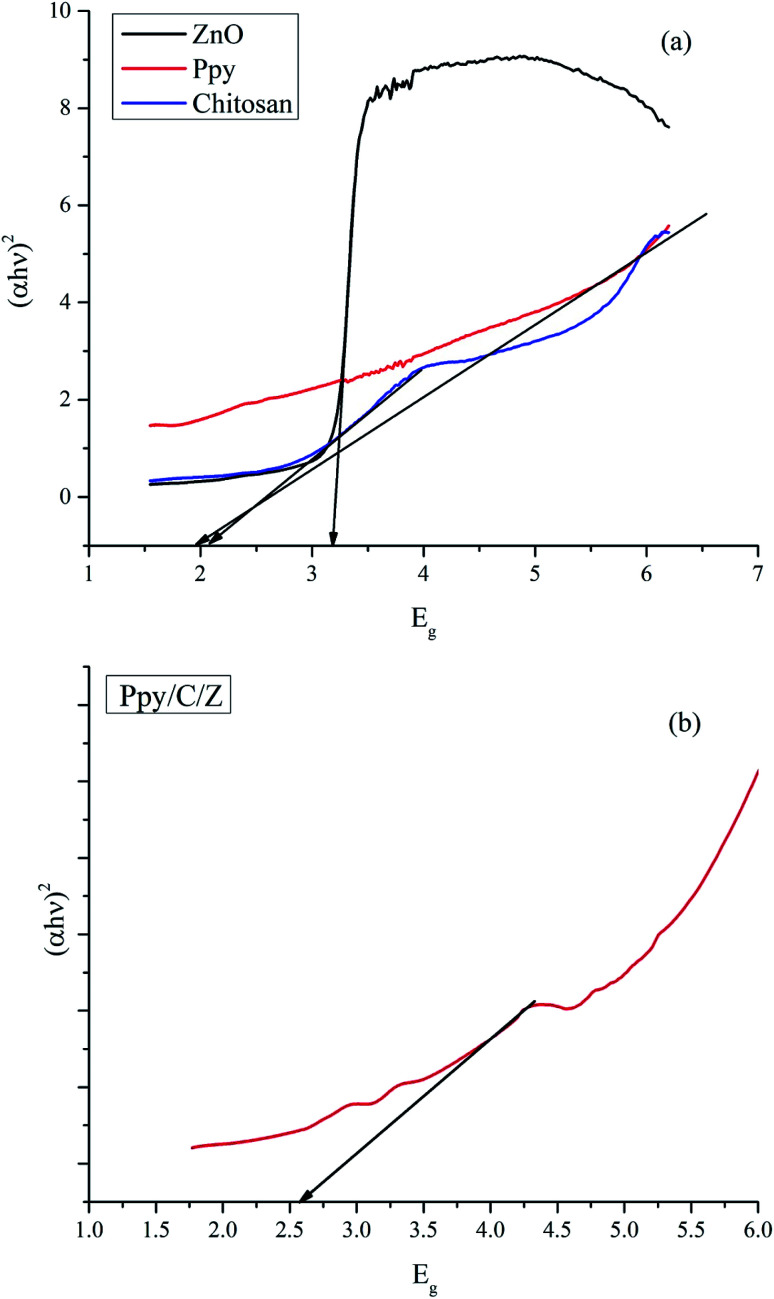
UV-DRS spectra and the bandgap calculation of (a) ZnO, chitosan and polypyrrole and (b) Ppy/C/Z bio-nanocomposite.

The optical properties of the as-synthesized ZnO, chitosan, Ppy and Ppy/C/Z nanoparticles were investigated using UV-diffuse reflectance spectroscopy (UV-DRS), as shown in Fig. 5. The bandgap of the as-prepared nanoparticles as well as the polymer matrices were calculated using the Kubelka Munk Function using the formulae (3) and (4):

3 (*hνα*) = (*Ahν* − *E*_g_)^*n*/2^

wherein, if *α*, the proportionality constant, is taken as the Kubelka–Munk function *F*(*R*), then the expression becomes

4 (*hνF*(*R*)) = (*Ahν* − *E*_g_)^*n*/2^

where *ν* is the frequency of light, *F*(*R*) is the Kubelka Munk function, *A* is the proportionality constant and *E*_g_ is the bandgap energy. The value of *n* can be determined by the type of optical transition: *n* = 1 for direct transition and *n* = 4 for indirect transition.^39,49,50^ The calculated bandgap values for ZnO, chitosan, Ppy and Ppy/C/Z are 3.3 eV, 2.1 eV, 1.9 eV, and 2.7 eV respectively. The bandgap value for ZnO is quite comparable to that reported elsewhere.^49,51^

### 3.6. Determination of electrochemical surface area

**Fig. 6:**
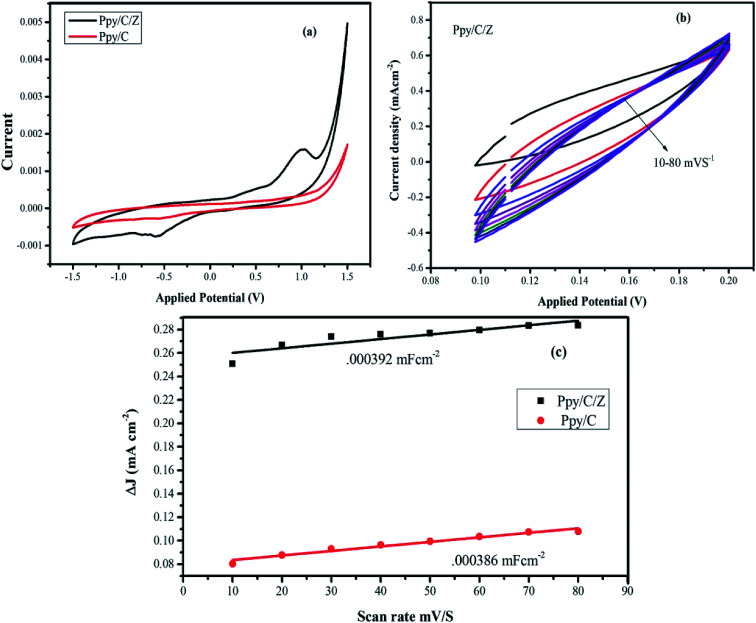
(a) Cyclic voltammograms of the nanocomposite materials; (b) EDLC curve of the Ppy/C/Z bio-nanocomposites at 10–80 mV s^−1^; and (c) calculated ECSA of the nanocomposites materials.

The electrochemical surface area was calculated by determining electrochemical double layer capacitance (EDLC) values using the cyclic voltammetry (CV) technique^52–54^ (see ESI†). The voltammograms were obtained in the potential range from −1.5 to 1.5 V at different scan rates, namely, 10 mV s^−1^, 20 mV s^−1^, and 30 mV s^−1^. EDLC values were determined from the CV curve (Fig. 6a), and then determined at different scan rates ranging from 10 to 80 mV s^−1^ (Fig. 6b). ECSA was obtained from the slope of EDLC and found to be 0.000386 mF cm^−1^ and 0.000392 mF cm^−1^, for Ppy/C and Ppy/C/Z, respectively (Fig. 6c). The calculated surface area is in good agreement with the activity of the photocatalyst. Higher surface area favours better adsorption of the dye on the photocatalyst surface due to existence of more reaction sites, which in turn promotes degradation.^55,56^

### 3.7. Probable mechanism of photocatalytic activity and kinetics of photodegradation

**Fig. 7:**
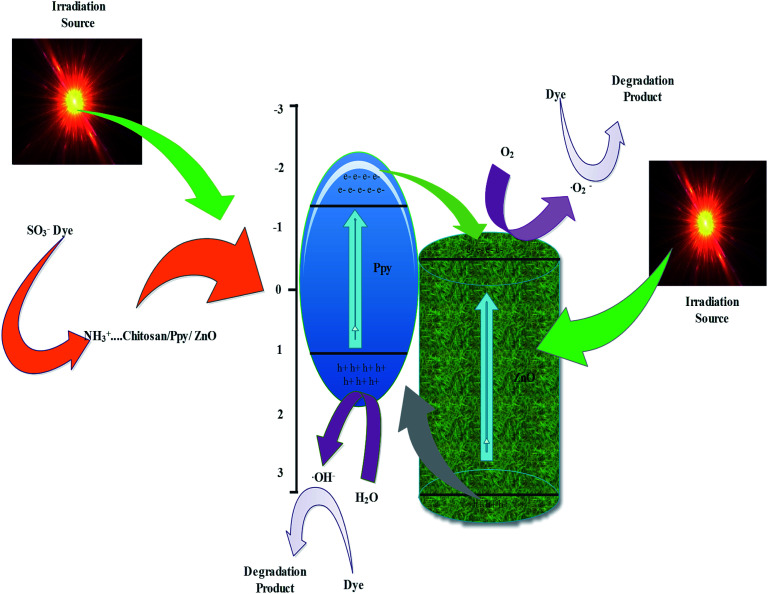
Probable mechanism of the photodegradation of dyes over the Ppy/C/Z photocatalyst.

**Fig. 8:**
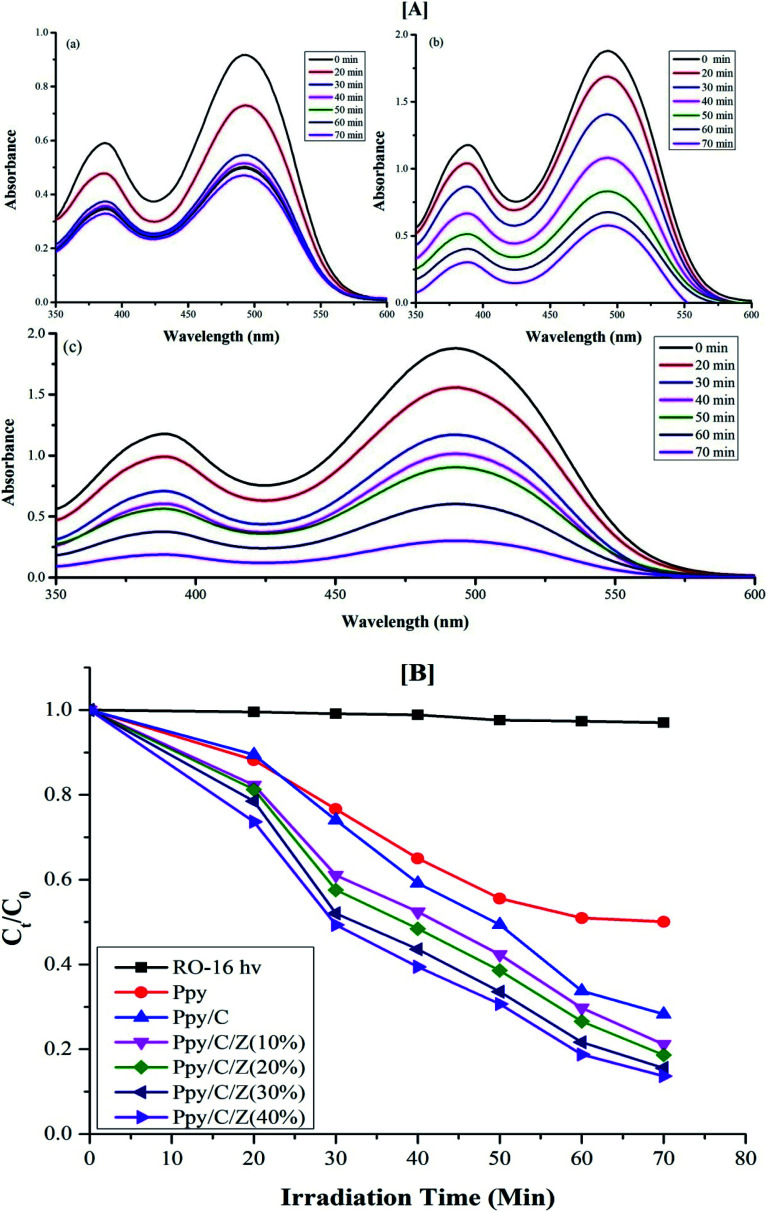
[A] UV-Visible spectra of degraded aliquots of RO-16 dye over (a) Ppy (b) Ppy/C (c) Ppy/C/Z (40%) and [B] spectra of the kinetics of photodegradation ability of the various catalysts.

Photodegradation of reactive orange-16 was performed in a photochemical reactor, as described in the experimental section. In the first step, the dye was adsorbed on the surface of the catalyst mainly due to the interaction of the SO_3_^−^ group of the reactive orange-16 dye with the protonated amine group of the chitosan. In the second step, upon UV light irradiation, electrons get excited from the valence band to the conduction band, leaving behind holes in the VB of ZnO; simultaneously, the electrons get excited from HOMO of the Ppy to the LUMO of Ppy. The electrons from the less positive LUMO of Ppy will prefer to flow down to the more positive CB of ZnO. Furthermore, the holes from the more positive VB of ZnO will prefer to flow into the less positive HUMO of Ppy. In this way, there will be an effective electron–hole separation. The electrons from the CB of ZnO will combine with O_2_ molecules to form superoxide radicals. Similarly, the holes from HOMO of Ppy will directly or indirectly mineralize the reactive orange-16 dye into simpler products. The complete mechanism is shown in Fig. 7. The UV-Visible spectra of the photodegraded aliquots of the reactive orange-16 dye by Ppy, Ppy/C and Ppy/C/Z are presented in Fig. 8A at the maximum absorbance at 493 nm. Further, to check the practical application of the photocatalyst, the photocatalysis study was extended and two more dyes, CBB-R-250 and MB, were used against the Ppy/C/Z catalyst. A significant degradation was obtained and the results of CBB-R250 and MB degradation are presented in Fig. 4S and 5S, respectively (ESI†). The degradation efficiency achieved was 85% of MB, 87% of RO-16 and 92% of CBB R-250 over the photocatalyst Ppy/C/Z (40%).

The valence and conduction band potentials of Ppy and ZnO were calculated using the following formulae ((5) and (6)):

5 *E*_VB_ = *X* − *E*^c^ + 0.5*E*_g_

where *X* is the electronegativity of the material expressed as the geometric mean of the electronegativity of the constituent element, *E*^c^ is the energy of the free electron on the hydrogen scale (4.5 eV) and *E*_g_ is the band gap energy.^38,57^ The *E*_VB_ of Ppy and ZnO was calculated and found to be 3.0 eV and 1.05 eV, respectively.^46^ The conduction band potential was calculated by the formula:

6 *E*_CB_ = *E*_VB_ − *E*_g_

The *E*_CB_ values were calculated to be −1.15 eV and −0.2 eV.

**Fig. 9:**
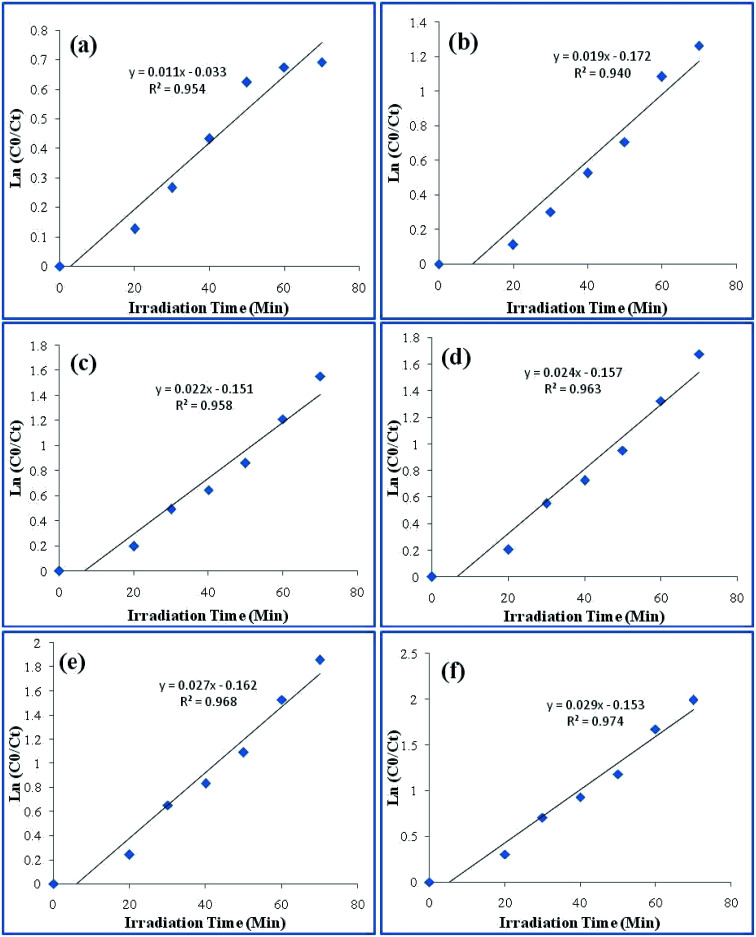
Pseudo first order rate constant curve of the various catalysts: (a) Ppy (b) Ppy/C (c) Ppy/C/Z (10%) (d) Ppy/C/Z (20%) (e) Ppy/C/Z (30%) and (f) Ppy/C/Z (40%).

The kinetics for the photodegradation of the nanocomposites was monitored using the following formula (eqn (7)) for the first order kinetics according to the Langmuir–Hinshelwood method7
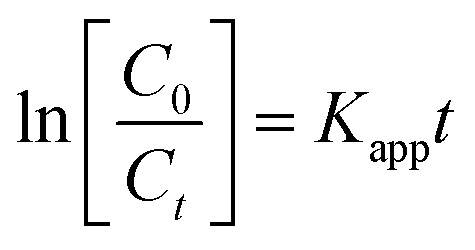
where *C*_0_ and *C* are the concentrations of the dye at time ‘0’ and ‘*t*’, respectively, and ‘*K*_app_’ is the apparent pseudo first order rate constant. The kinetics of photodegradation is given in Fig. 8B. The rate constant for the degradation of the dye for Ppy/C and Ppy/C/Z (40%, 30%, 20%, and 10%), Ppy and blank reactive orange-16 in the presence of UV light was found to be 0.0253 min^−1^, 0.0232 min^−1^, 0.0209 min^−1^, 0.01941 min^−1^, 0.01579 min^−1^, 0.01014 min^−1^ and 0.00037 min^−1^, respectively, and the pseudo first rate constant plot is presented in Fig. 9.

**Fig. 10:**
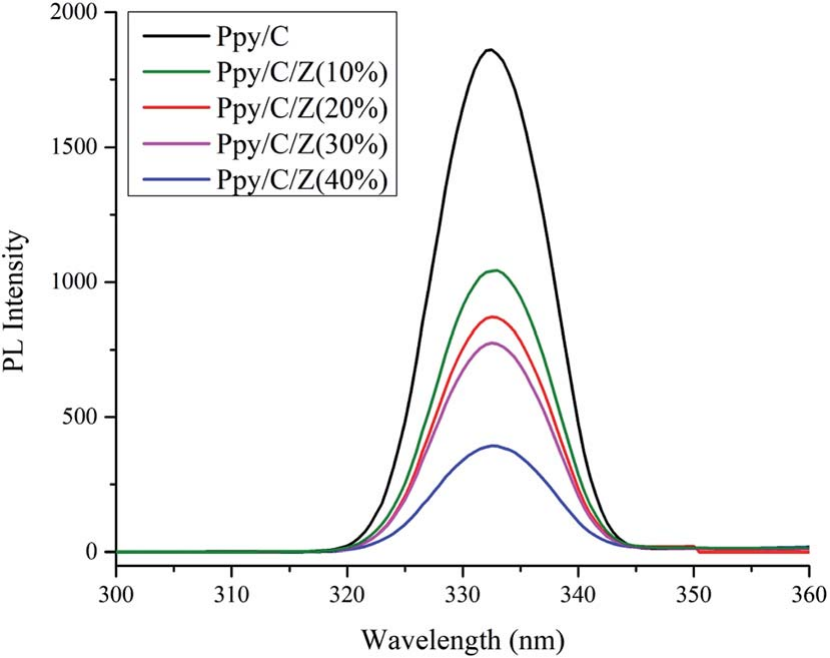
Photoluminescence spectra of the various photocatalysts used in the photodegradation.

Photoluminescence spectra are based on the recombination rate of the charge carrier species upon irradiation, and the recombination rate of the electron hole pairs resulted in the PL intensity.^38^ Thus, the PL intensity is directly proportional to the recombination rate. As it can be seen in Fig. 10, photoluminescence spectra of the photocatalyst Ppy/C, and bionanocomposites with different weight percent of ZnO with Ppy/chitosan, *i.e.*, Ppy/C/Z (10%), Ppy/C/Z (20%), Ppy/C/Z (30%), and Ppy/C/Z (40%) have been obtained *via* fluorescence spectroscopy. From the spectra, it is clearly evident that the highest intensity peak for Ppy/C is due to the high recombination rate of HOMO and LUMO, and high recombination rate causes less photocatalytic activity. In contrast, in case of the Ppy/C/Z catalyst, decreased PL intensity results from the low recombination rate of the electron–hole pairs, resulting in better photodegradation.

### 3.8. Study of reactive species and stability of the bio-nanocomposites

**Fig. 11:**
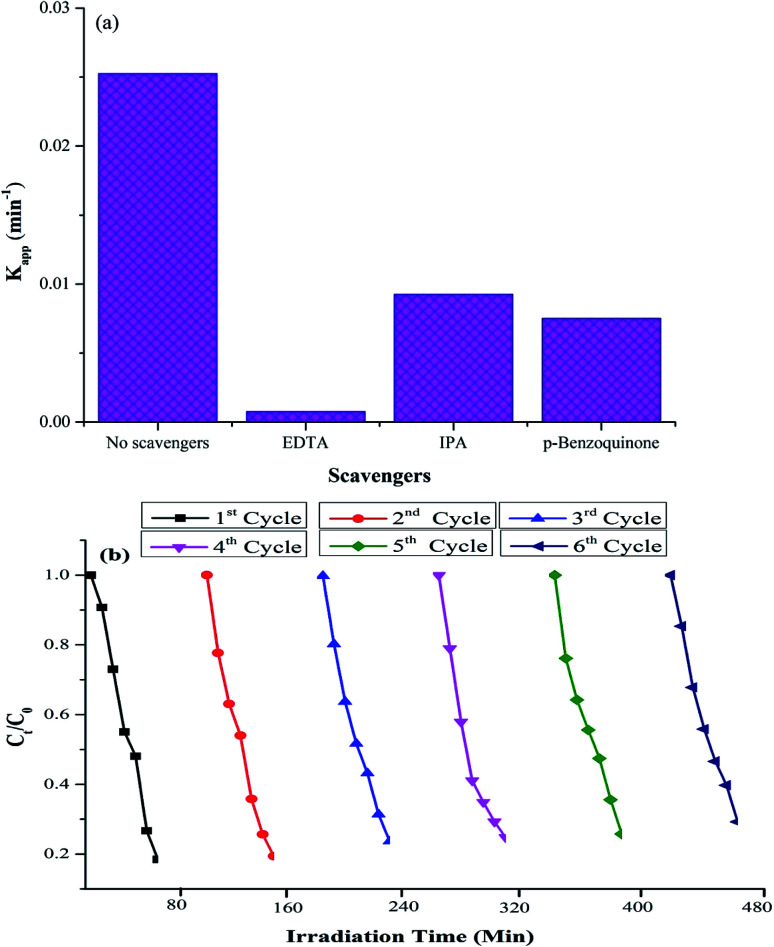
(a) Effects of different scavengers on photodegradation and (b) recycling experiments of the photocatalytic degradation of dyes.

To identify the reactive species involved in the photocatalysis, various scavangers, namely, *p*-benzoquinone, disodium ethylenediaminetetraacetate (EDTA) and isopropyl alcohol (IPA) were used as trapping agents to quench ·O_2_^−^, h^+^ and ·OH, respectively.^57^ The effects of different scavengers on the photodegradation are presented in Fig. 11a. The decrease in the *K*_app_ value upon the addition of trapping agents to quench ·O_2_^−^, h^+^ and ·OH shows that all the trapping agents have significant effects on the photodegradation. However, the significant changes in the degradation process upon the addition of EDTA to quench h^+^ indicate that holes are the primary reactive species involved in the photocatalytic oxidation of the RO-16 dye under the influence of Ppy/C/Z.

Further, to check the stability of the photocatalyst, the experiments were repeated for up to six consecutive cycles. Fig. 11b shows the recycling experiment of Ppy/C/Z against the RO-16 dye. The percent degradation of the dye was around 87%, 80.6%, 77%, 75% 72% and 71% from 1^st^ to 6^th^ cycle, respectively. Thus, it can be concluded that the photocatalyst remains stable even after the 6^th^ cycle. The photocatalyst did not lose its activity significantly up to six cycles.

### 3.9. Electrochemical impedance spectroscopy (EIS) study and COD test

**Fig. 12:**
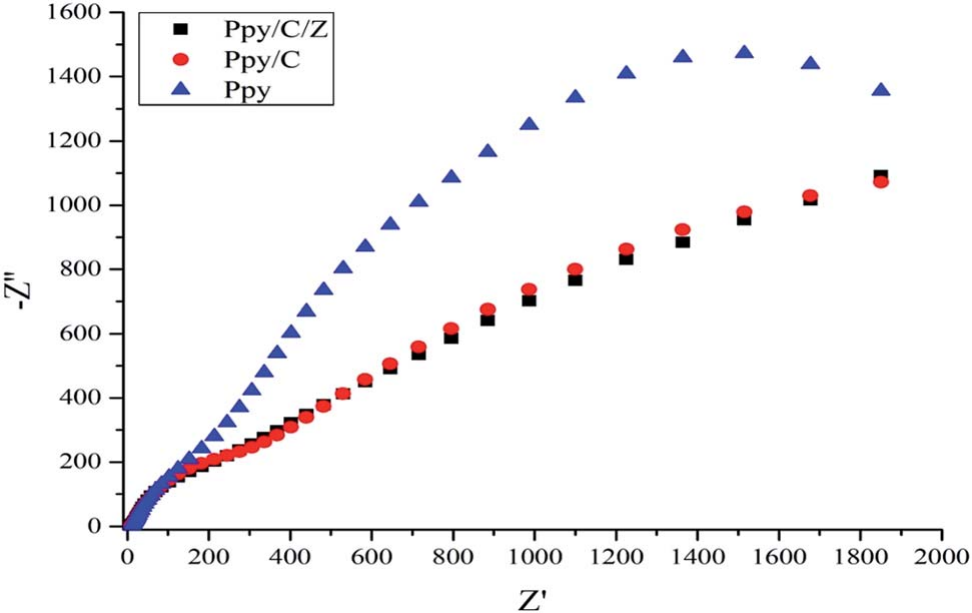
Nyquist plots of the Ppy, Ppy/C and Ppy/C/Z photocatalysts.

To further examine the enhanced photocatalytic activity of the as-prepared nanocomposites, the decoupling efficiency of the e^−^ and hole pairs should be studied. Electrochemical impedance spectroscopy has been performed to study the EIS Nyquist plots.^58,59^ It is clear for the Fig. 12 that the arc radius of the Nyquist plot of Ppy/C/Z is smaller than that of Ppy/C and pure Ppy, which suggests that in the Ppy/C/Z composites, the charge separation of the photogenerated electrons and holes is higher as compared to that in Ppy/C and Ppy. The COD test was performed according to our previous literature report^60^ to confirm the mineralization of the RO-16 aliquots under the influence of the Ppy/C/Z photocatalyst. The percentage removal efficiency based on the chemical oxygen demand was found to be 29.3%. It was observed that the COD of the aliquots calculated from photodegradation at 10 min intervals showed a decreasing trend and the plots are presented in Fig. 6S (ESI†).

### 3.10. Enhanced *in vitro* antibacterial activity of the as-prepared Ppy/C/Z bio-nanocomposites

**Fig. 13:**
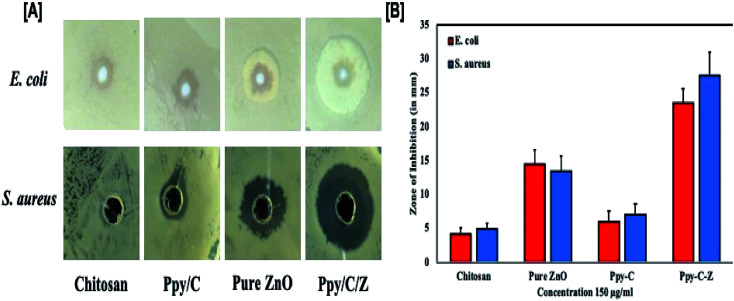
Antibacterial potential of the as-synthesized nanocomposites: [A] pictorial representation of the zone of inhibition of one of the three experiments and [B] the histograph showing the diameter of the zone of inhibition (in mm) against *E. coli* and *S. aureus* microbial strains after treatment with various formulations.

*In vitro* results of the agar well diffusion susceptibility test revealed that the Ppy/C/Z bio-nanocomposite showed enhanced bactericidal activity as compared to Ppy/C and untreated cells. The Ppy-C-Z bio-nanocomposite showed minimum inhibitory concentrations of 64 mg ml^−1^ and 32 mg ml^−1^ against *S. aureus* and *E. coli*, respectively. Moreover, it was observed that the Ppy/C/Z bio-nanocomposite has admirable bactericidal potential against potent Gram-negative as well as Gram-positive pathogenic strains, and 128 mg ml^−1^ and 64 mg ml^−1^ of the Ppy-C-Z bio-nanocomposite was found to be the minimum bactericidal concentration against *S. aureus* and *E. coli*, respectively. The zone of inhibition results, as represented in Fig. 13, clearly demonstrated the potent bactericidal effect of the Ppy-C-Z bio-nanocomposite at the concentration of 0.15 mg ml^−1^. Post treatments with various formulations, the zone of inhibition results are presented in Fig. 13.

Chitosan has polycationic amines that can interact with the Gram-negative *E. coli* bacteria through the negatively charged residues of proteins, lipids and carbohydrates anchored on the bacterial surface that consequently restrict the bacterial growth. In addition, chitosan can easily interact with the teichoic and teichuronic acids, which are polysaccharides found within the cell wall of Gram-positive bacteria such as *S. aureus*, which results in a sequence of events that ultimately leads to bacterial death. In addition to this, ZnO induces oxidative stress in the bacterial strains by permeation into the cells that eventually results in the restriction of bacterial growth and ultimately leads to death.

The results further suggested the therapeutic importance of the biodegradable Ppy/C/Z bio-nanocomposite against bacterial pathogens (Gram-positive as well as Gram-negative), as revealed by the zone of inhibition and CFU assay. Additionally, the results of the present study put forward the wide spectrum anti-bacterial activity of the Ppy/C/Z bio-nanocomposite against the deadly pathogens *S. aureus* and *E. coli*.

### 3.11. Enhanced cytotoxic effect of Ppy/C/Z bio-nanocomposites against cervical and breast cancer cells

**Fig. 14:**
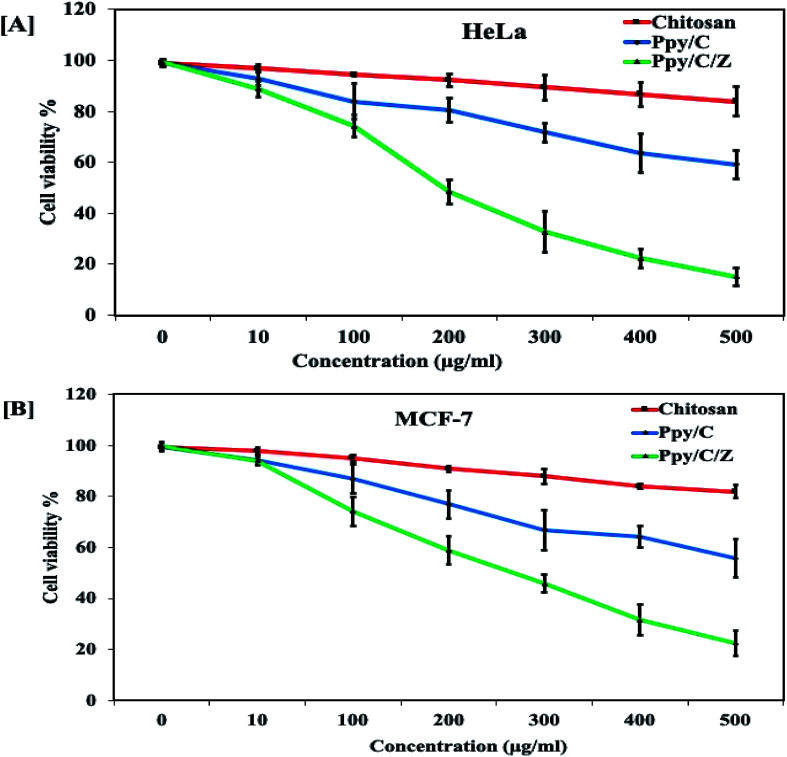
Cytotoxicity profile of Ppy/C and Ppy/C/Z on cancer cell lines.

Anticancer potential of Ppy/C and Ppy/C/Z was evaluated following the standard MTT assay. Briefly, cells were plated in a flat bottom 96-well plate at a density of 5 × 10^4^ cells per well. After incubation for 24 h at 37 °C, the culture medium was replaced with 100 μl fresh medium containing serially increasing concentrations of various formulations. Post treatment, cells were incubated for 48 h and cell viability was measured by adding 20 μl of the MTT dye (5 mg ml^−1^ in phosphate-buffered saline) per well. The plates were incubated for a further period of 4 h and thus, formazan crystals formed due to the reduction of the dye by viable cells in each well were dissolved in 200 μl dimethyl sulfoxide (DMSO). The absorbance was recorded in an ELISA plate reader at 570 nm. The absorption values were expressed as percent cell viability compared with the untreated control group considered as 100% viable. The result of the MTT assay demonstrated that the Ppy-C-Z bio-nanocomposite showed an enhanced cytotoxic potential against various human cancer cell lines when compared with the other forms, as shown in the Fig. 14.

**Fig. 15:**
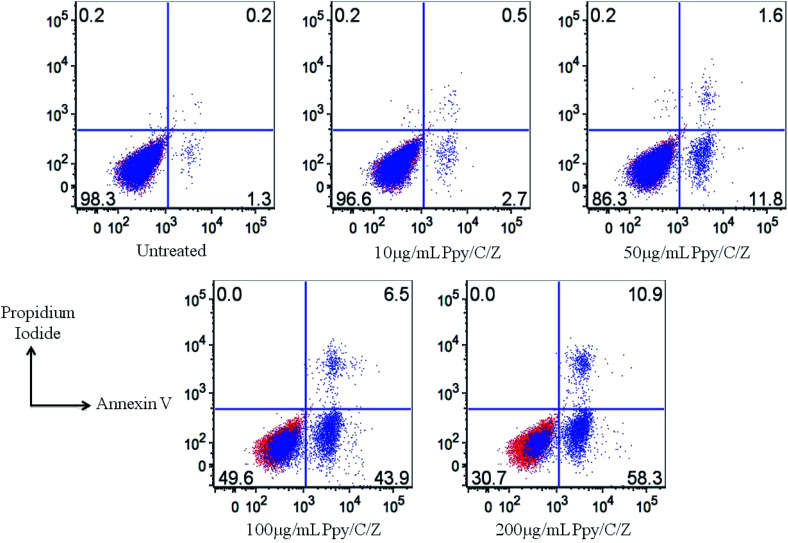
Determination of apoptosis induction by analysing the apoptotic cell population by Annexin V and Propidium Iodide (PI) staining with the treatment using varying concentrations of the Ppy/C/Z bio-nanocomposites, as indicated. The FACS data showed the significant increase in the population of early and late apoptotic cells with the increase in the concentration of the bio-nanocomposite from 10 μg ml^−1^ to 200 μg ml^−1^.

Dose-dependent cytotoxicity was induced by Ppy/C and the Ppy/C/Z bio-nanocomposite. The cytotoxicity was found to be more enhanced in the Ppy/C/Z bio-nanocomposite-treated cells of the HeLa cervical cancer cell line [A] as well as in the MCF-7 breast cancer cell line [B]. Further, the apoptosis data against the HeLa cells were acquired on the flow cytometer on treatment with varying concentrations of the Ppy/C/Z bionanocomposite, as shown in Fig. 15, to provide more insight. Our bio-nanocomposite showed an astonishing cytotoxic potential against the cancer cells with the increase in concentration, as depicted from the total annexin V positive cells as compared to the untreated cells.

From the above data, the therapeutic potential of the biodegradable Ppy/C/Z bio-nanocomposite has been clearly revealed by the antibacterial assay performed against bacterial pathogens (Gram-positive as well as Gram-negative) as well as by zone inhibition and CFU assay. Furthermore, the data proposed the wide spectrum anti-bacterial effect of the Ppy/C/Z bio-nanocomposite on the Gram-positive *S. aureus* strain as well as on the Gram-negative *E. coli* strain. Likewise, the novel formulation prepared in this study showed a promising result against cancer cell lines without harming healthy tissues. As a matter of fact, chitosan has free amine groups, which not only offer high positive surface charge, but also allow it to easily interact with the cell membrane (negatively charged).^61^ This property of chitosan enhances the cellular uptake of the Ppy/C/Z bio-nanocomposite by the altered target cells.

## 4. Conclusion

The as-prepared bio-nanocomposites have been used as photocatalysts for the degradation of MB, RO-16 and CBB R-250 dyes. From the results, it can be concluded that upon the incorporation of the different weight percentage of ZnO nanoparticles to Ppy/C, the degradation rate increased. A maximum of 85% of MB, 87% of RO-16 and 92% of CBB R-250 can be degraded by the bionanocomposite containing 40 weight percent of ZnO. The electrochemical surface area of Ppy/C/Z was found to be higher, which in turn leads to higher activity of photodegradation. Using the ternary compound as the photocatalyst, the rate of transfer of electrons also increased, resulting in the formation of more reactive species. Holes, superoxide radicals and hydroxide radicals are responsible for the photodegradation. However, holes are the primary reactive species in the photodegradation, and the photocatalyst was found to be quite stable even after six cycles. ZnO nanoparticles have been effective against tested strains, whereas chitosan/ZnO is quite effective against HeLa cervical cancer line and MCF-7 breast cancer cell line. From the data, it has been observed that Ppy/C/Z shows effective antibacterial and cytotoxic properties. Moreover, apoptosis data showed the enhanced apoptotic potential of the bio-nanocomposite against cancer cells, as acquired on the flow cytometer. Percentage of the apoptotic cells increases with the increase in the concentration of Ppy/C/Z bio-nanocomposites from 10 to 200 μg ml^−1^. Keeping the above results into consideration, it can be concluded that the Ppy/C/Z bio-nanocomposite can be applied for wastewater treatment and has augmented antibacterial and anti-cancer potential.

## Conflicts of interest

There are no conflicts of interest to declare.

## Supplementary Material

RA-009-C9RA06493A-s001
