# Supplementary material for: Zinc oxide-decorated polypyrrole/chitosan bionanocomposites with enhanced photocatalytic, antibacterial and anticancer performance
Source: RSC Adv. 2019 Dec 12;9(70):41135–50. doi: 10.1039/c9ra06493a (PMC9076406; doi:10.1039/c9ra06493a)
Supplement: RA-009-C9RA06493A-s001 [file RA-009-C9RA06493A-s001.pdf]

Electronic Supplementary Material (ESI) for RSC Advances.
This journal is © The Royal Society of Chemistry 2019

Supplementary Information

In the present study ECSA was calculated from electrochemical double layer capacitance method as reported earlier [52-54] by plotting the graph between scan rates and current density. The current obtained from the CV curve was changed to current density using the following formula.

$$Current\ density = \frac{Current * 1000}{Area\ of\ glassy\ carbon\ electrode}$$

Where, area of 5 mm glassy carbon electrode is 0.19625 m^2.

Now we calculate the electrochemical double layer capacitance (C_{dl}) from the following equation:

$$\nu = \Delta J\ C_{dl}$$

Where ν is the scan rates and C_{dl} is the linear slope equivalent to twice the C_{dl} was used to represent the ECSA.

A picture is shown below (Figure 1S) representing the complete circuit to perform cyclic voltammetry.

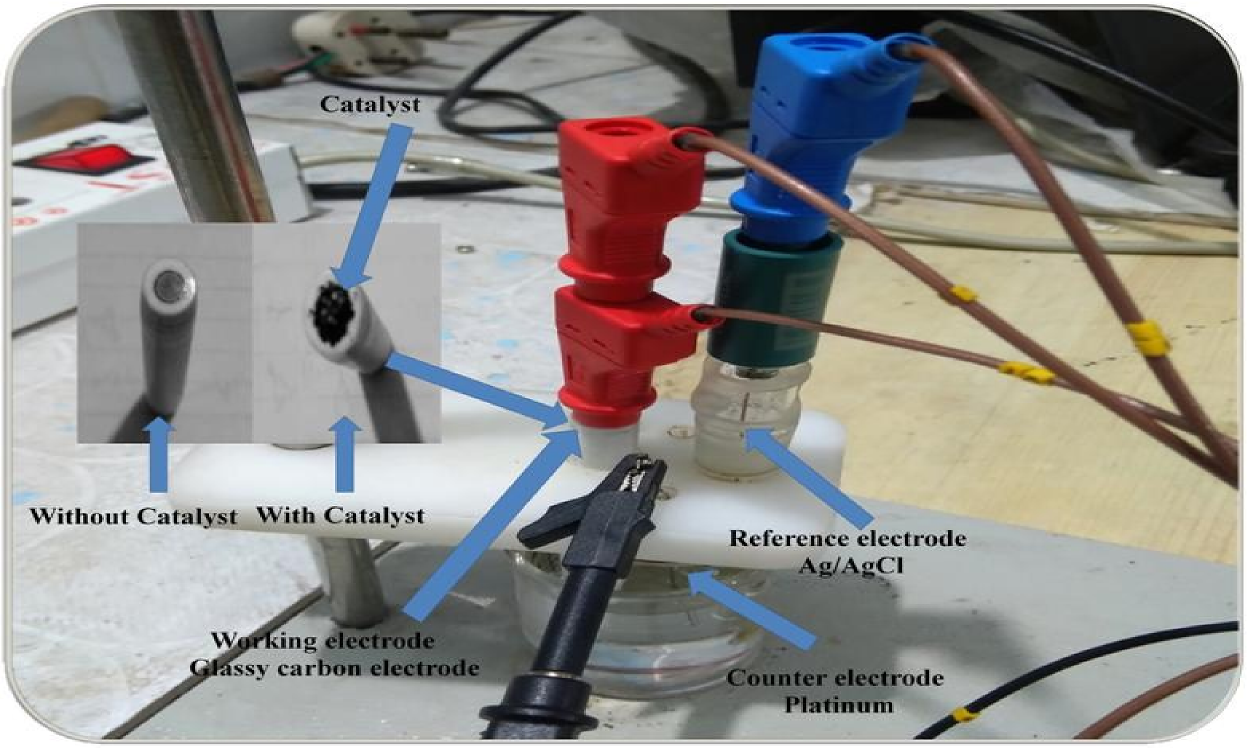

Fig. 1S Picture represents the circuit for the cyclic voltammetry performed.

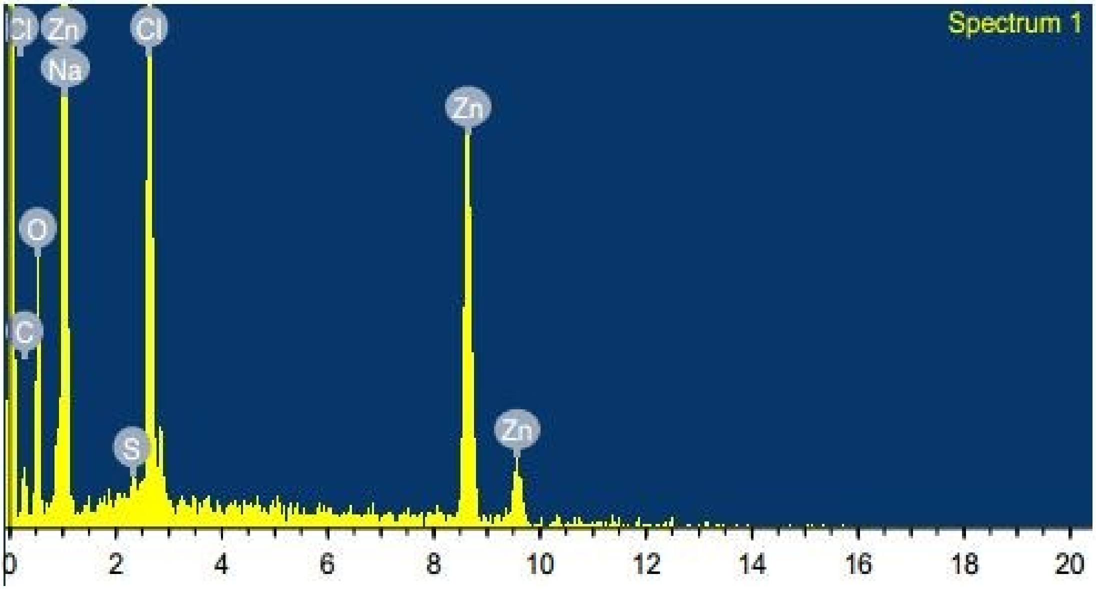

Fig. 2S EDX images to show the elemental composition of the as prepared bio-nanocomposite.

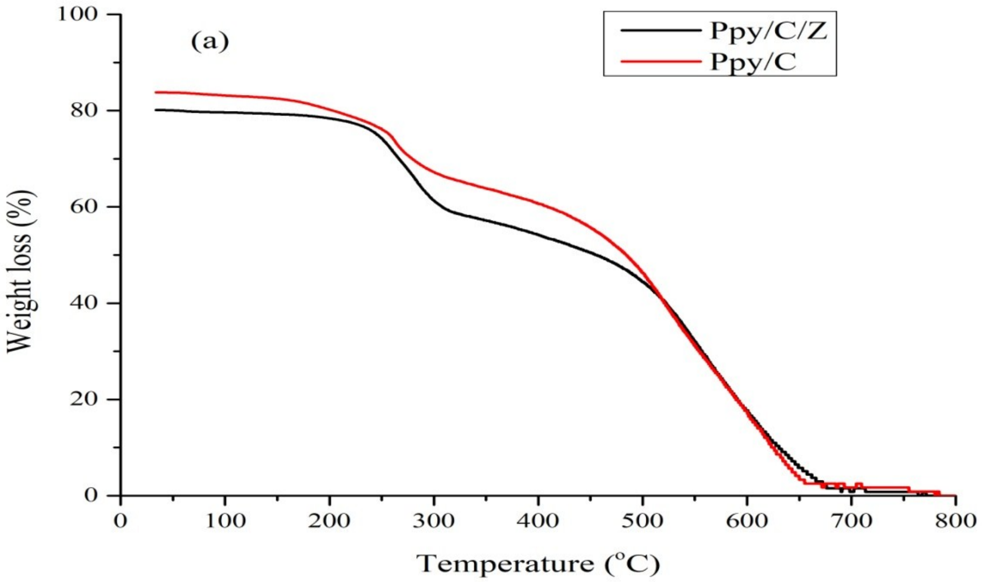

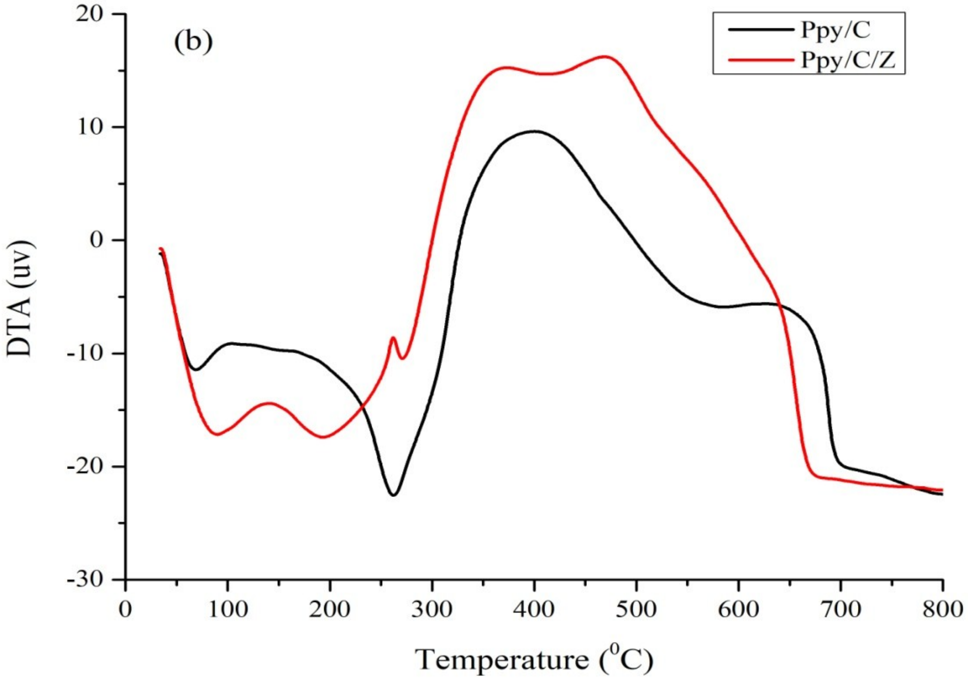

Fig. 3S TGA and DTA spectra of the as prepared nanocomposites materials.

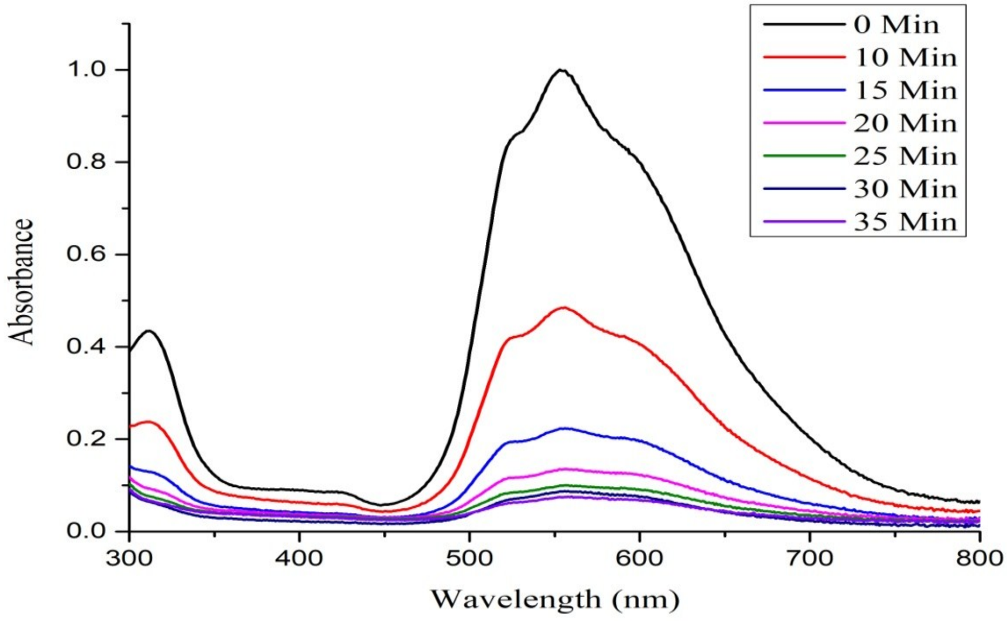

Fig. 4S UV-visible spectra of the photodegradation of CBB-R-250 dye over the Ppy/C/Z photocatalyst.

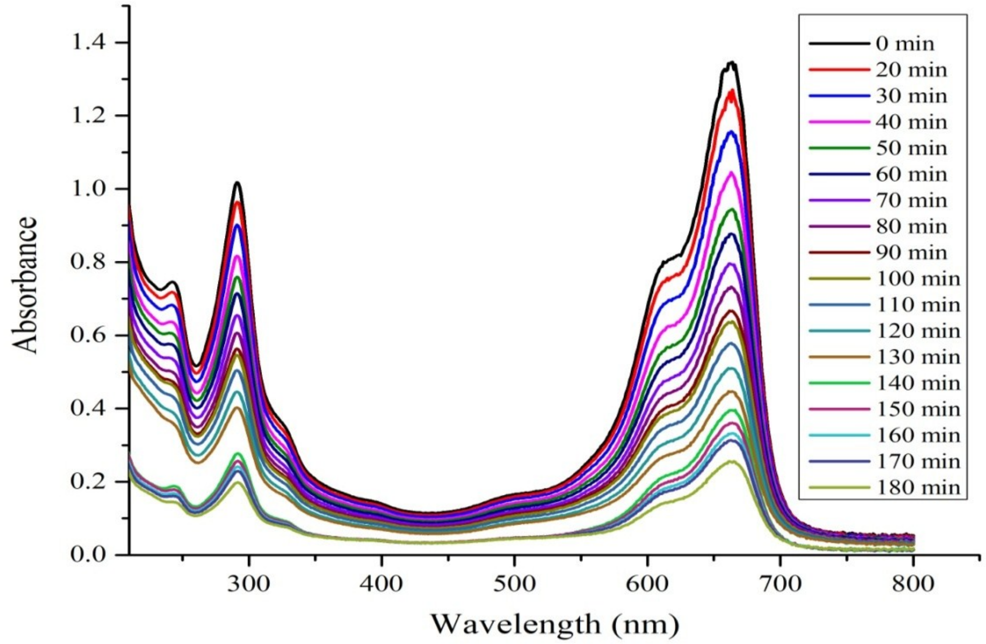

Fig. 5S UV-visible spectra of the photodegradation of MB dye over the Ppy/C/Z photocatalyst.

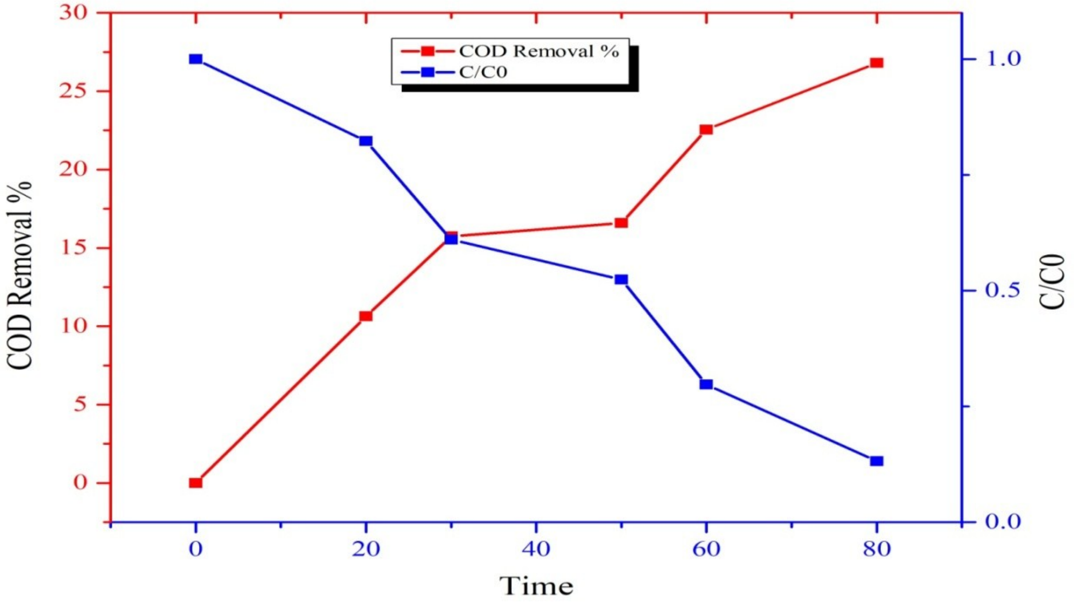

Fig. 6S COD curve of the degraded aliquots.
